# Aligning Biomedical Research With Neurodiversity to Support the Metabolic Health of Autistic Individuals

**DOI:** 10.1002/osp4.70117

**Published:** 2026-01-28

**Authors:** Emily Hotez, A. Janet Tomiyama

**Affiliations:** ^1^ Department of General Internal Medicine‐Health Services Research The University of California, Los Angeles (UCLA) David Geffen School of Medicine Los Angeles California USA; ^2^ Department of Psychology The University of California, Los Angeles (UCLA) Los Angeles California USA

**Keywords:** autism, biomedical research, neurodiversity, obesity

## Abstract

Autistic individuals represent approximately 1 in 31 people in the United States and experience disproportionately high rates of obesity, type 2 diabetes, cardiovascular disease, and feeding and eating challenges, alongside reduced life expectancy. However, evidence‐based metabolic health interventions for autistic populations remain sparse. This Perspective synthesizes evidence on two interconnected barriers that limit metabolic health research in the autism field: (1) lack of accessible biomedical research methodologies and (2) insufficient attention to mechanisms underlying poor metabolic health in this population, including chronic stress and weight stigma. Drawing on principles from neurodiversity, Universal Design for Research, and the Academic Autism Spectrum Partnership in Research and Education (AASPIRE) guidelines, we outline a neuro‐affirming paradigm that can improve metabolic health research in the autism field. Finally, we provide phase‐by‐phase practical recommendations for researchers, spanning study design, measure development, recruitment, consent, screening, data collection, and interpretation. Aligning metabolic health research with neuro‐affirming principles can generate more rigorous, representative, and ethically grounded evidence and ultimately support more meaningful improvements in metabolic health and overall well‐being for autistic individuals across the life course.

## 1 Introduction

Autistic individuals represent 1 in 31 people in the United States. Compared with their non‐autistic counterparts, they experience disproportionate rates of poor metabolic health, with greater incidence of type 2 diabetes, cardiovascular disease, central obesity, and feeding and eating challenges (e.g., food and sensory aversions and disordered eating attitudes). This population also experiences premature mortality, with an average 16‐year lower life expectancy [1, 2, 3]. These findings highlight the need to identify modifiable risk factors and develop corresponding metabolic health interventions for autistic populations.

Metabolic health interventions designed for autistic individuals remain sparse and of low quality. As an example, a meta‐analysis of 12 weight management intervention studies found only one “high‐quality” study, few with sub‐group analyses and female participants, and only half reporting positive results [4]. The reviewed studies featured physical activity interventions (*n* = 4), pharmaceutical interventions (specifically focusing on metformin [*n* = 2]), and “comprehensive” interventions (*n* = 6), which included nutrition, physical activity, and motivational components (e.g., opportunities for social interaction, goal setting, and family involvement).

Interventions remain limited, in part, due to two interrelated barriers in research involving autistic populations: [1] a lack of accessible biomedical research methodologies and [2] the low prioritization of studies examining the biomedical mechanisms underlying poor metabolic health. This manuscript describes these research barriers that limit the evidence base and outlines opportunities to adopt a *neuro‐affirming paradigm* that prioritizes well‐being, autonomy, and meaningful research participation.

## 2 Barrier: Lack of Accessible Research Methodologies

Accessibility in research is multi‐faceted and includes: (1) *physical accessibility* (e.g., sensory‐friendly environments and accommodations for motor or communication differences); (2) *intellectual accessibility* (e.g., plain language materials, visual supports, and low‐literacy options); and (3) *social accessibility* (e.g., culturally respectful framing, non‐stigmatizing language, and trauma‐informed practices) [5].

Biomedical research is frequently physically, intellectually, and socially inaccessible for autistic individuals, who often experience heightened stress, anxiety, and discomfort related to research participation [6, 7]. From a physical accessibility perspective, in‐person laboratory visits commonly required in biomedical research can pose challenges for autistic participants, as clinical settings are often associated with sensory overstimulation [8], transportation barriers [9], and unfamiliar, unpredictable environments [10]. These barriers may be further intensified using physically stressful procedures, including blood draws [11].

From an intellectual accessibility perspective, research materials often rely on language that is overly complex or laden with technical jargon. Informed consent forms, for example, frequently fail to use plain language [12]. Similarly, data collection materials often rely on vague or imprecise wording, which autistic participants report makes it difficult to respond accurately. For example, studies often ask participants to report on their health or lifestyle behaviors; however, autistic individuals report that such questions frequently fail to capture the substantial variability in their daily experiences and they are challenged to respond accurately [13, 14].

Finally, from a social accessibility perspective, autism research has long prioritized identifying the biomedical underpinnings of autism [15]. This emphasis has often carried implicit—and at times explicit—assumptions that the goal of such research is to “cure” or “eradicate” autism, contributing to widespread and understandable mistrust of biomedical research among autistic individuals. This mistrust is further compounded by the field's reliance on a traditional medical model of disability and a deficit‐focused research paradigm [16], particularly when studies appear to prioritize causes or genetic risk over outcomes that meaningfully improve quality of life. Many well‐intentioned biomedical studies fail to acknowledge this historical context, rendering research practices potentially stigmatizing or even traumatic for autistic participants.

Furthermore, lack of accessibility in autism research often requires researchers to rely primarily on non‐autistic caregiver or proxy report measures, even when autistic individuals can reliably provide self‐report data beginning in childhood [17]. The incorporation of validated self‐report measures for autistic individuals—particularly for subjective phenomena such as stress and body image—is necessary. Omitting self‐report data not only limits participants' agency but also omits subjective experiences—such as internalized weight stigma or stress (see below)—that may be critical antecedents and consequences of poor metabolic health in this population.

Finally, limited researcher transparency about study goals has further discouraged autistic adults from participating in research—especially biomedical studies—alongside concerns about data misuse and confidentiality [6, 18, 19]. Together, these factors can potentially reduce the motivation to participate in research among this population. The consequences of research inaccessibility are both ethical and scientific. Ethically, inaccessible protocols limit participants' ability to make informed choices and exercise autonomy. Scientifically, lack of accessibility in research can compromise research quality by leading to low enrollment, participant withdrawal, and incomplete data.

## 3 Barrier: Lack of Prioritization of the Underlying Mechanisms of Poor Metabolic Health

In non‐autistic populations, a well‐established “vicious cycle” describes multiple pathways through which chronic stress contributes to maladaptive eating, weight cycling, and fat storage [20]; however, few studies in autism have examined these underlying mechanisms to poor metabolic health. Autistic individuals face unique chronic stressors, including lifelong stigma, sensory overwhelm, and social exclusion [21]. Simultaneously, scoping reviews find that disordered eating attitudes and behaviors are significantly more prevalent in autistic youth, relative to non‐autistic youth [3]. For example, some autistic children have been found to engage in “emotion‐linked over‐ and under‐eating,” with autistic girls experiencing more emotional over‐eating, compared to autistic boys [22].

Lack of prioritization of underlying mechanisms to poor metabolic health in autistic individuals may be related to challenges in assessing chronic stress biomarkers—such as cortisol, a key stress hormone—in this population. When cortisol is measured, studies often rely on invasive methodologies (e.g., blood draws or saliva collection), which can deter potential participants. Hair cortisol sampling is an example of an underutilized non‐invasive alternative that can capture cumulative stress exposure over a 3‐month period [23]. Broader adoption of non‐invasive methodologies for obtaining biomarkers may facilitate additional metabolic health studies in autism.

The low prioritization of the relationship between chronic stress and metabolic health in autistic individuals has resulted in limited examination of weight stigma as a chronic stressor in this population. In the general population, weight stigma contributes to poor health and mental health outcomes by promoting disordered eating, stress, and avoidance of medical care [23]. Among autistic youth and adults, weight stigma is rarely acknowledged as a contributor to poor metabolic health, despite its potential role as an environmental driver of adverse health outcomes. For example, autistic participants have reported that weight‐related discussions with healthcare providers can provoke anxiety [24]. A recent scoping review of eight studies that investigated body image and autism identified potential associations between measures of negative body image and autistic traits, suggesting that weight stigma in healthcare may be particularly damaging to this population [25].

As a result of these gaps in the research, obesity may be incorrectly attributed exclusively to individual factors—rather than to both environmental and biological factors—in autistic populations. Pathologizing both autism and weight may disempower autistic individuals and their families from participating in metabolic health research.

## 4 Toward a Neuro‐Affirming Paradigm

To overcome these barriers, the field can adopt a *neuro‐affirming paradigm* that prioritizes well‐being, autonomy, and meaningful participation. Neuro‐affirming research is grounded in the concept of *neurodiversity,* which draws on the social model of disability and recognizes autism as a form of human diversity, rather than as a pathology. Several theoretical and methodological frameworks—including Universal Design for Research [26] and The Academic Autism Spectrum Partnership in Research and Education (AASPIRE) Guidelines [27, 28]—outline principles and strategies for implementing neuro‐affirming research.

### 4.1 Inclusion and Accessibility

Neuro‐affirming studies are designed to promote the inclusion of autistic individuals as both participants and partners in the research process [27]. This can be accomplished through intentional study design principles that facilitate community engagement, remove arbitrary exclusion criteria, provide accommodations for participation, and apply Universal Design to all research materials and protocols [26].

### 4.2 Respect for Lived Experience

Neuro‐affirming research entails maintaining an open dialog among researchers and autistic individuals throughout the lifetime of a study. Indeed, it is increasingly recognized that lived and experiential expertise is equally as valuable as professional expertise in shaping high‐quality research [29]. Qualitative research reveals that many autistic research participants welcome opportunities to engage in and co‐produce research [30], including co‐design, advisory boards, focus groups, and other participatory methodologies.

### 4.3 Transparency and Trust‐Building

Researchers can maximize transparency in the research process, which can build trust with autistic participants and communities. In practice, this entails communicating the study purpose, risks, and benefits using plain language in the consent process; embedding greater transparency in the recruitment so participants have more information about the study prior to enrollment; and sharing results back with participants and the broader autism community in accessible formats [31].

### 4.4 Capabilities Over Individual Deficits

A capabilities approach—a widely cited developmental framework—has recently been applied to research and practice for autistic individuals [32]. This approach de‐emphasizes individual abilities and underscores the importance of *opportunities* that can be facilitated or constrained by contextual and environmental factors. In practice, applying a capabilities approach to metabolic health diverts focus towards systems and supports—not individuals—that can be modified or improved to promote thriving, resilience, and quality of life [28]. For example, addressing weight stigma in healthcare acknowledges the systemic and structural factors that lead to downstream metabolic health consequences.

A capabilities approach also shifts the focus away from predetermined and standardized intervention outcomes and emphasizes that health can take many different forms based on individual, family, and community goals. As a result, research that aligns with a capabilities approach would incorporate a strengths‐based approach that recognizes the abilities and assets of autistic individuals and their families, rather than solely focusing on deficits or challenges. Research shows that a strengths‐based approach can improve societal perceptions of autism and reduce internalized stigma among autistic individuals [33].

## 5 Practical Recommendations for Researchers Studying Autism and Health

Table 1 provides an overview of adaptation researchers can make to each phase of a study, in alignment with neuro‐affirming principles. These principles and strategies are described below.

**TABLE 1:** Neuro‐affirming research principles and example strategies, organized by research phase^a^.

Phase/Principle	Example strategies
Study design	
Accommodate participants' needs throughout research activities [26].	Do not exclude participants with disabilities unless their inclusion would fundamentally change the study's scientific validity. Consult disability experts to ensure the project is accessible.
Create research instruments and instructions in multiple formats so participants can access the information [26].	Offer auditory, visual, tactile, and plain‐language options for communicating essential information. Make all participant materials (e.g., consent forms, instruments, intervention instructions) available in multiple formats and allow each participant to select their preferred format. Ensure study materials are technically accessible. Prepare print materials in formats compatible with the National Instructional Materials Accessibility Standard [34]. Ensure study websites are screen‐reader accessible and ADA‐compliant.
Use proxy reporters only when direct participation is impossible, even with accommodations and supports [27].	Create a separate proxy survey, adapting items to distinguish what the proxy can accurately report for the participant versus what reflects the proxy's own perspective.
Avoid focusing solely on autistic individuals' weaknesses and challenges [28].	Conceptualize studies to capture autistic individuals' strengths and explore how these can be leveraged to support success and thriving. Examine how autistic individuals' environments, contexts, and social networks (e.g., school, family, peers) may create barriers or foster resilience and thriving. Assess the impact of discrimination and stigma on autistic individuals.
Acknowledge that research is not fully objective and that researchers' social positions—including being neurotypical—can introduce bias [28].	Be aware of potential biases and actively work to counter them when selecting research questions and designing studies, including by engaging community advocates.
Engage diverse community stakeholders—including autistic individuals and their parents—in research decisions to reduce bias and increase relevance [28].	Promote autistic participation in research by forming community advisory boards and using community‐based participatory research methods.
Acknowledge past research that has harmed or failed autistic people, and actively work to earn trust [28].	Recognize that researchers—not autistic participants—bear the responsibility for fostering reconciliation.
Measure development	
Do not assume that instruments validated with general populations, caregivers, or children are valid for autistic individuals [27].	Evaluate whether adaptations are needed; if so, modify the instrument and re‐test psychometric properties. Use a participatory process to assess, create, or adapt instruments. Add prefaces for clarity or context, and revise items to simplify sentences, remove passive voice, and clarify pronouns. Replace difficult vocabulary, confusing terms, or figures of speech with simpler language; if substitution is not possible, add definitions, examples, or clarifications. When response options are unclear, consider using graphics (e.g., partially filled cylinders, frowning/smiling faces) to improve clarity.
Design and administer assessments and data collection tools that are fully accessible [27].	Collaborate with community partners to ensure assessments are precise, contextually grounded, and adequately scaffolded. Use probes to anchor events and encourage elaboration.
Recruitment	
Offer multiple ways for people to learn about, respond to, and access opportunities to participate in research [26].	Use multiple media channels for recruitment, including local disability agencies and consumer organizations. Provide recruitment materials in multiple formats to reach diverse audiences. If you accept online responses, ensure the recruitment website is ADA‐accessible. Include contact information for requesting reasonable accommodations in all recruitment materials.
Consent	
Ensure the consent process is fully accessible [27].	Adapt consent forms by simplifying language, removing unnecessary barriers, adding images, and offering text‐to‐speech options for online forms. Partner with autistic individuals to co‐create more accessible consent materials. Reduce participant burden by offering online consent options.
Screening	
Minimize undue influence and exploitation while promoting autonomy and inclusion [27].	Do not automatically require decisional capacity assessments for individuals with an autism diagnosis; consider the study's risk level and the types of decisions participants routinely make. If decisional capacity is uncertain, use an accessible consent process followed by a brief comprehension assessment.
Data collection	
Whenever possible, offer multiple participation modes to include autistic participants with diverse strengths and needs [27].	Use software with read‐aloud capability to support participants with low literacy. Provide both synchronous and asynchronous participation options, and allow oral or written communication (e.g., email, phone, in‐person, instant messaging).
Design and implement data collection tools and assessments that are fully accessible [27].	Offer participants the option to review materials in advance. Begin data collection with a clear preface explaining the type of responses desired. Use concrete, specific questions rather than abstract prompts.
Use proxy reporters only when direct participation is not possible, even with accommodations [27].	Distinguish between a *supported participant* (participant answers with assistance) and a *proxy* (supporter answers with minimal participant input). Provide supporters with a separate mechanism to share their own perspectives.
Acknowledge that research is influenced by researchers' social positions—including being neurotypical—which can introduce bias [28].	Use language that avoids negative value judgments and favors neutral or positive terms when describing autistic individuals.
Acknowledge past research that has harmed or neglected autistic people, and actively work to rebuild trust [28].	If harm has occurred, openly acknowledge it and validate community concerns. Demonstrate commitment to listening and responding to the community through concrete actions.
Anticipate participants' needs during data collection and research activities [26].	For sessions lasting 2 hours or more, schedule planned breaks or rest periods.
Interpreting and translating findings	
Acknowledge that research is shaped by researchers' social positions and backgrounds—including being neurotypical—which can introduce bias [28].	Interpret findings from multiple perspectives, considering whether results reflect strengths, neutral differences, or disabling environmental factors rather than individual deficits.
Learn about the ideas, theories, and concepts autistic people use to interpret and understand their own experiences [28].	Consider how these perspectives may reshape interpretation of findings and generate new research questions.

^a^ Examples are derived from the publications cited for each principle.

## 6 Study Design and Development of Research Materials

The first step in making metabolic health research neuro‐affirming is to hire and train research teams that understand and value neurodiversity. This can be accomplished by explicitly stating in hiring and training materials that neurodiversity is valued and providing training on bias reduction, active listening, and accessibility. Such training programs are underway, with participants reporting positive experiences [35]. These trainings can also occur beyond the research study itself to promote a culture of neurodiversity across academic and research institutions [36]. An even more effective long‐term strategy may involve integrating stigma‐prevention efforts into primary and secondary school curricula [37].

Neuro‐affirming research teams are well‐equipped to design and implement neuro‐affirming studies. At the study‐design stage, researchers can ensure that each exclusion criterion aligns with a scientific rationale [26]. Specifically, instead of imposing additional exclusion criteria, researchers can maximize accessibility and opportunities for inclusion by providing accommodations that facilitate participation [26]. These principles also apply to measure development. As an example, researchers may offer participants options to contribute data using multiple modalities (e.g., survey vs. interview) and ensure all materials are written in plain language. Additionally, measures validated solely with non‐autistic populations may not be appropriate for autistic participants [27]; measures should be pilot‐tested and adapted as necessary or new measures should be developed.

## 7 Recruitment, Consent, Screening, and Data Collection

Neuro‐affirming research can become more accessible, inclusive, and strengths‐based by maximizing opportunities for autistic individuals—not only supporters or proxies—to actively participate. Applying Universal Design frameworks, commonly used in educational settings, can help create inclusive research environments where diverse individuals can fully engage. Research processes can be made multimodal, including how individuals learn about study opportunities, communicate with research staff, interact with data collection materials, and participate in interventions [26].

Autism researchers have established guidance on when proxy reporting is appropriate. In general, proxy reporting should be used only when direct participation is not possible, even with accommodations and supports [27]. When decisional capacity is uncertain, the AASPIRE guidelines recommend using an accessible informed consent process followed by a brief comprehension assessment. If proxy reporting is required, researchers should provide clear instructions to proxies and ensure that data are interpreted appropriately [38].

Additionally, researchers can make studies more accessible to autistic individuals by offering remote, flexible, and low‐burden data collection options [39], including validated, non‐invasive biomarker sampling. The “lab‐in‐a‐box” model—where participants collect biomarker data from home—has been implemented in neurotypical populations and has shown promising preliminary results with autistic participants [40, 41]. However, these models need to be rigorously evaluated and scaled for the populations most likely to benefit. If recruitment or data collection occurs virtually, researchers can align web‐based platforms with accessibility standards [42].

## 8 Interpreting and Translating Findings

Despite efforts to remain objective, research is inevitably shaped by researchers' social positions and backgrounds, which can introduce bias [28]. For example, researchers have noted high levels of disordered eating behaviors in autistic individuals—particularly in autistic women—including food selectivity, mealtime rigidity, and other eating difficulties [43, 44]. However, these behaviors may arise from different underlying factors than in non‐autistic populations—such as stress regulation or sensory sensitivities—rather than weight management. Furthermore, disordered eating behaviors may be simultaneously adaptive (e.g., food selectivity to manage sensory issues) and maladaptive (e.g., food restriction due to weight and shape concerns) for autistic populations [45]. Therefore, collaboration with the autistic community in measure development, data collection, and interpretation is essential to ensure that study conclusions are accurate and contextually meaningful.

## 9 All Stages of the Research Process

Participatory methodologies, which allow autistic individuals to actively contribute to research, can strengthen adherence to all principles of neuro‐affirming research. According to Arnstein's Ladder of Citizen Participation, participatory methodologies can range from minimal participation to full power‐sharing [46]. While equal co‐production can make autism research maximally relevant to the autistic community, it is not always feasible given resource constraints. Evidence shows that less intensive consultation models—such as autistic advisory boards—can still improve the quality and relevance of studies when designed using neuro‐affirming principles [28, 47]. Even when participatory approaches are limited, researchers can enhance engagement by sharing findings with participants, inviting feedback and offering meaningful opportunities for contribution.

Community partnerships are essential for effective implementation of participatory methodologies. Many of the recommendations proposed in this manuscript derive from the work of AASPIRE, which includes representatives from academic, self‐advocate, family, and professional communities [48]. AASPIRE offers concrete strategies for effective community partnerships in autism, including methods for inclusive shared decision‐making in research partnerships. Academic and research collaboratives seeking to promote more inclusion in research are emerging beyond autism research (e.g., in mental health more broadly) [49]. Greater integration of these strategies in metabolic health research is needed.

Additionally, researchers can practically implement a strengths‐based approach to promoting metabolic health for autistic individuals by reframing research questions focused on narrowly defined metrics (e.g., obesity based on the body mass index [BMI]) toward promoting metabolic health and overall well‐being and adapting outcomes to reflect individual and family health goals [33].

Researchers can also accomplish this by using non‐stigmatizing language related to both autism and weight [50, 51]. Researchers can proactively address weight stigma by using sensitive terminology and framing weight within a holistic well‐being context. Autistic researchers have set forth concrete guidance for non‐stigmatizing autism language [51]. Participatory research approaches can further support this effort by involving individuals with lived experience and partnering with organizations that serve people with intellectual and developmental disabilities [39].

## 10 Conclusion

By integrating neuro‐affirming approaches, researchers can generate more rigorous, representative, and impactful evidence on metabolic health. These approaches not only advanced science but can also lead to more meaningful improvements in the health and well‐being of autistic individuals across the life course.

## Funding

This work was funded by National Institute of Diabetes and Digestive and Kidney Diseases (Grant no 1K01DK140611‐01A1).

## Disclosure

The authors used Chat GPT‐4 to assist in condensing the word count and streamlining the manuscript to meet journal requirements. All content was subsequently reviewed, revised, and verified for accuracy. The authors accept full responsibility for the integrity and accuracy of this manuscript.

## Conflicts of Interest

The authors declare no conflicts of interest.

## Data Availability

The authors have nothing to report.
